# Reduction of Ca^2+^ Entry by a Specific Block of KCa3.1 Channels Optimizes Cytotoxic Activity of NK Cells against T-ALL Jurkat Cells

**DOI:** 10.3390/cells12162065

**Published:** 2023-08-15

**Authors:** Miguel Olivas-Aguirre, Laura Hadit Cruz-Aguilar, Igor Pottosin, Oxana Dobrovinskaya

**Affiliations:** 1 Laboratory of Immunobiology and Ionic Transport Regulation, University Center for Biomedical Research, University of Colima, Colima 28045, Mexico; molivas@ucol.mx (M.O.-A.); laguilar3@ucol.mx (L.H.C.-A.); 2 Division of Exact, Natural and Technological Sciences, South University Center (CUsur), University of Guadalajara, Guzmán City 49000, Mexico

**Keywords:** acute lymphoblastic leukemia, Jurkat cells, NK cells, NK-92, NK-mediated killing, KCa3.1 channel, intracellular calcium, store-operated calcium entry

## Abstract

Degranulation mediated killing mechanism by NK cells is dependent on store-operated Ca^2+^ entry (SOCE) and has optimum at moderate intracellular Ca^2+^ elevations so that partial block of SOCE optimizes the killing process. In this study, we tested the effect of the selective blocker of KCa3.1 channel NS6180 on SOCE and the killing efficiency of NK cells from healthy donors and NK-92 cells against T-ALL cell line Jurkat. Patch-clamp analysis showed that only one-quarter of resting NK cells functionally express KCa3.1 current, which increases 3-fold after activation by interleukins 15 and 2. Nevertheless, blockage of KCa3.1 significantly reduced SOCE and intracellular Ca^2+^ rise induced by IL-15 or target cell recognition. NS6180 (1 μM) decreased NK degranulation at zero time of coculture with Jurkat cells but already after 1 h, the degranulation reached the same level as in the control. Monitoring of target cell death by flow cytometry and confocal microscopy demonstrated that NS6180 significantly improved the killing ability of NK cells after 1 h in coculture with Jurkat cells and increased the Jurkat cell fraction with apoptotic and necrotic markers. Our data evidence a strong dependence of SOCE on KCa3.1 activity in NK cells and that KCa3.1 specific block can improve NK cytotoxicity.

## 1. Introduction

Natural killer (NK) cells are in the first line of the antitumor defense and share killing mechanisms with cytotoxic T lymphocytes (CTL). The dominant mode of the NK and CTL serial killing activity is mediated by the exocytosis of lytic granules containing perforin and granzyme molecules [1]. Polarization of lytic granules with respect to target cells does not depend on Ca^2+^, whereas degranulation strongly depends on Ca^2+^ entry via calcium release-activated channel (CRAC), as clearly demonstrated for NK cells from patients with strong genetical defects in CRAC components as well as after pharmacological suppression of CRAC in NK from normal subjects [2]. Contact of CTL and NK cells with the target ones induces a cytosolic Ca^2+^ ([Ca^2+^]_i_) increase in effector cells, which triggers the release of cytotoxic granules [3,4,5,6]. The difference between CTL and NK cells is the activation mechanism. CTL is activated via major histocompatibility complex-dependent antigen presentation to the T cell receptor, whereas NK is via a set of activation receptors on the cell surface when they outbalance the impact of inhibitory ones. Both pathways converge at the level of phospholipase C, whose activity produces inositol 1, 4, 5 triphosphate, causing activation of its receptor channel and Ca^2+^ release from the endoplasmic reticulum, whose depletion induces the assembly and activation of CRAC [7]. There is evidence coming mainly from in vitro studies by Markus Hoth’s group that NK and CTL killing activity is a non-monotonic function of external and internal Ca^2+^ and has a relatively low [Ca^2+^]_i_ optimum, close to the resting [Ca^2+^]_i_ level: whereas almost total suppression of Ca^2+^ entry prevents degranulation, too high [Ca^2+^]_i_ may result in an exaggerated lytic granules release, which will therefore primarily target cells in the close proximity of effector ones, hence, with a lesser overall efficiency in time [6,7,8,9]. Of note, Ca^2+^ sensor protein Munc13-4, a critical regulator of exocytosis in mast, CTL, and NK cells, displays a non-monotonic Ca^2+^ dependence, with an optimum close to that observed for killing by CTL and NK [10]. It has been proposed then that partial pharmacological suppression of the CRAC-mediated Ca^2+^ influx (store-operated Ca^2+^ entry, SOCE) may be beneficial for the NK and CTL antitumor activity [8].

CRAC acts as a major Ca^2+^ entry channel in most non-excitable cells, so its blockage can have severe collateral effects. CRAC-mediated Ca^2+^ influx ceases with depolarized voltages due to inward rectification of CRAC current and a decrease of Ca^2+^-driving force [11], whereas in excitable cells, expressing voltage-dependent Ca^2+^ channels, the depolarization provokes a progressive channels’ opening, resulting in an increased Ca^2+^ influx. This fact underlies a differential interaction of Ca^2+^ activated K^+^ channels (KCa) with Ca^2+^ channels in excitable and non-excitable cells: as KCa channels are activated by elevated Ca^2+^ and mediate K^+^ efflux, they will move the membrane potential towards equilibrium potential for K^+^, causing a hyperpolarization. Such voltage shift will turn off voltage-dependent Ca^2+^ channels in excitable cells but, in contrast, ensures a sustained Ca^2+^ influx by CRAC in non-excitable cells [12]. KCa3.1, which is widely expressed in non-excitable tissues (various blood cells, mast cells, microglia, epithelial and endothelial cells, and fibroblasts), is a voltage-independent channel, steeply activated by a moderate increase of cytosolic Ca^2+^ above the resting level [13,14]. CRAC and KCa3.1 cooperate with each other so that inflowing Ca^2+^ activates the KCa3.1 channel, and KCa3.1 activity ensures membrane hyperpolarization to maintain Ca^2+^ influx by CRAC sufficiently high to keep KCa3.1 in the open state [15]. This general mechanism is widely accepted [13,14,16,17], despite somewhat limited direct experimental evidence. For T lymphocytes, the mutual interaction between SOCE and KCa3.1 is supported by several original studies [18,19,20,21]. However, a single available work on B lymphocytes showed that, albeit KCa3.1 activity hyperpolarized the cell, it had no significant influence on the Ca^2+^ entry [19]. No pertinent information is available for NK cells.

CRAC-mediated Ca^2+^ influx underlies many lymphocyte functions, including proliferation, interleukin secretion, formation of the immunological synapse, and migration [17]. There are two K^+^-selective channels in human lymphocytes, already mentioned KCa3.1 and voltage-dependent Kv1.3 channel. Kv1.3 channel dominates in resting B and T lymphocytes and can potentially support CRAC, albeit it has a limited repolarization capacity, as these channels are closed completely at voltages below −60 mV [22]. In activated T and B cells, where the KCa3.1 channel is strongly upregulated, the function of the latter becomes pivotal, and the role of Kv1.3 diminishes [23].

**Figure 1:**
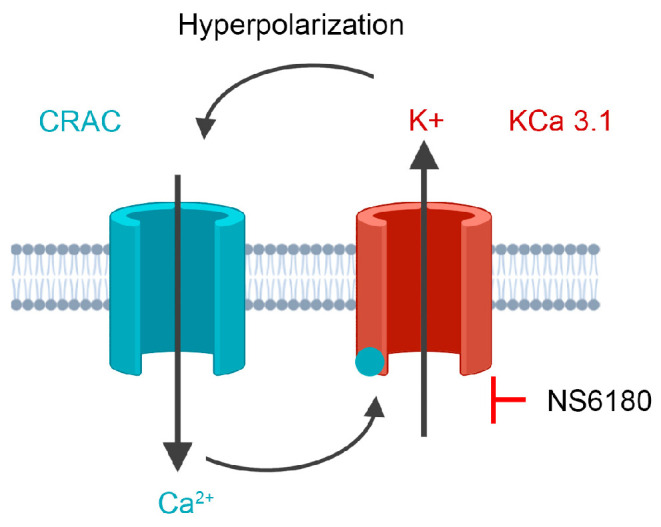
The experimental strategy is based on cooperative interactions between CRAC and KCa3.1 channel. CRAC mediates an increase of cytosolic Ca^2+^, which activates KCa3.1. K^+^ efflux via KCa3.1 provokes membrane hyperpolarization, hence relatively high sustained Ca^2+^ influx by CRAC. Disruption of this interaction by the selective block of KCa3.1 should reduce CRAC activity and global Ca^2+^ signal. See text for details.

Much less is known about the functional roles of K^+^ channels in NK cells. A complete non-specific block of K^+^ channels in NK cells or induced membrane depolarization impairs their killing activity against leukemic K562, U937, Molt-4, and lymphoma Daudi and Raji cell lines [24,25]. Koshy and co-workers [26] applied specific blockers of KCa3.1 and Kv1.3 channels to primary human NK cells and found out that the Kv1.3 block either had no effect or inhibited the proliferation and degranulation of NK cells, whereas KCa3.1 block increased degranulation and killing, but did not affect migration, expression of chemokine receptors and formation of conjugates with target cells. In light of the aforementioned considerations of KCa3.1 functional interaction with CRAC, SOCE-dependent degranulation, and the existence of the optimal [Ca^2+^]_i_ concentration for killing by NK, it is logical to propose that KCa3.1 channel block would reduce SOCE (Figure 1), thus optimizing degranulation and the killing process. To address this hypothesis, we used a selective KCa3.1 blocker NS6180 to check whether it reduces the IL-15 and target cells (T-ALL cell line Jurkat) induced [Ca^2+^]_i_ signal and SOCE in NK cells and whether KCa3.1 block affects NK degranulation and killing of Jurkat cells. We used both NK cells from healthy donors and the human NK-92 cell line, with a high antitumor cytotoxicity and a high potential for chimeric antigen receptor (CAR) therapy [27].

## 2. Materials and Methods

### 2.1. Reagents

All the reagents, commercial kits, and solvents employed within this article are summarized in Appendix A. The highest tested NS6180 concentration was 10 μM. The respective solvent (DMSO) concentration was 0.1%; in no test, 0.1% of DMSO provoked any significant effect.

### 2.2. Cell Lines and Culture Conditions

NK-92 (CRL-2407) and Jurkat clone E6-1 (TIB-152) were from the American Type Culture Collection (ATCC; Manassas, VA, USA). All growth media and supplements were purchased from Gibco, Thermo Fisher Scientific (Fair Point, NY, USA). Jurkat cells (male, 14 years) were grown in suspension in advanced RPMI 1640 medium, supplemented with 5% (*v*/*v*) of heat-inactivated fetal bovine serum (FBS), 100 U/mL of penicillin, 100 µg/mL streptomycin, and 1% of GlutaMAX™. NK-92 cells were grown in αMEM media without ribonucleosides (Gibco, 12561-056), supplemented with β mercaptoethanol (Sigma-Aldrich, M3148; 100 μM), inositol (200 μM), folic acid (20 μM), horse serum and fetal bovine serum (12.5% *v*/*v*, respectively) and interleukin 2 (IL-2; 400 UI/mL). Optimal IL-2 concentration was determined experimentally to maintain cell culture healthy, as was evidenced by increased cell number, aggregate formation, and the minimum presence of dead cells. IL-2 stock solution (1 µg/mL in sterile water) was prepared from lyophilized product (Stemcell, 78,036.2), aliquoted (40 μL), and stored at −80 °C. Fresh complete medium was prepared every 48 h with the addition of freshly thawed aliquot IL-2, and an NK-92 subculture procedure was performed, avoiding disruption of cell aggregates to prevent loss of cell viability. For experiments, several cell aggregates were separated, gently resuspended, washed, and subjected to corresponding experimental procedures. All cell lines were used for experimentation during the first 20 culture passages and maintained in a humidified incubator at 37 °C with 5% CO_2_.

### 2.3. Isolation and Purification of Primary NK Cells

Fresh, unrefrigerated whole blood was obtained from healthy volunteers. In total, the data from 2 females and 2 males, aged 20 to 35 years, were analyzed. Each of them was given a brief explanation of the aims of the study and signed the informed consent in accordance with the Declaration of Helsinki. The protocols were approved by the Bioethics and Biosecurity Committee of the Biomedical Research Centre and the Faculty of Medicine of the University of Colima (Approval Number 2020-07), in agreement with the federal laws (Artículo 100, Ley General de Salud). All procedures were performed in a laminar flow biosafety cabinet (class II), ensuring the integrity of the samples and the operator´s protection. The whole blood was diluted 1:1 with cold PBS and centrifuged over Ficoll Histopaque-1077 (Cytiva, 17144003); 1:1.5 at 1500× *g* for 30 min at 4 °C. Then, PBMCs were washed with PBS and resuspended in complete RPMI-1640. Cells were then rested overnight at 37 °C with 5% CO_2_. For NK isolation, the *NK Cell Isolation Kit* (Miltenyi Biotec, 130-092-657) was used according to the manufacturer’s specifications. NK cell (CD3^−^CD56^+)^ purity was evaluated by flow cytometry (FACS Canto II cytometer, BD Biosciences, Franklin Lakes, NJ, USA). Cells were stained simultaneously with the anti-CD3 antibody conjugated with PE, and the anti-CD56 antibody conjugated with FITC (Biolegend, 300308 and 362546, respectively); under these conditions, a purity of 96% was obtained (Appendix B). Primary NK cells were cultured up to 72 h in advanced RPMI 1640 medium, supplemented with 5% (*v*/*v*) of heat-inactivated fetal bovine serum (FBS), 100 U/mL of penicillin, 100 µg/mL streptomycin, and 1% of GlutaMAX™, in a humidified incubator at 37 °C with 5% CO_2_.

### 2.4. Electrophysiology

Experiments were performed using the whole-cell configuration of the patch-clamp technique, employing the Axopatch 200A amplifier (Axon Instruments, Seattle, WA, USA). Patch pipettes were fabricated from Kwik-Fil 1B150F-4 capillaries (World Precision Instruments, Sarasota, FL, USA), using a multistep pipette puller Brown/Flamming model P-97 (Sutter Instruments, Novato, CA, USA) and polished using a microforge (List Medical, Germany). Patch electrodes were filled with a solution containing 130 mM KCl, 0.5 mM CaCl_2_, 2 mM HEDTA (free Ca^2+^ 1 μM, calculated using winmaxc32 program https://somapp.ucdmc.ucdavis.edu/pharmacology/bers/maxchelator/downloads.htm (accessed on 4 February 2023) and 10 mM of HEPES-KOH (pH 7.4). Patch electrodes have a resistance of 3–5 MΩ. The bath solution contained 160 mM KCl, 2.5 mM CaCl_2_, 10 mM glucose, and 10 mM HEPES-KOH (pH 7.4). High external K^+^ enhanced inward current by KCa3.1 and prevented the accumulation of Kv1.3 inactivation in a sequence of voltage ramps. Capacity and access resistance were measured by means of a patch-clamp amplifier, and compensation of 70–80% was applied for access resistance (typically in the range of 10–25 MΩ). Records were low pass filtered at 5 kHz, digitized using the DigiData 1200 interface (Axon Instruments, Foster City, CA, USA), transferred to a PC, and analyzed using the pClamp 6.0 program (Axon Instruments, Foster City, CA, USA). For pharmacological analysis, drugs were introduced by bath perfusion to the following final concentrations: highly potent Kv1.3 blocker margatoxin (MgTx 1 nM) and specific KCa3.1 blocker NS6180 (300 nM). Ionic currents were recorded under voltage-clamp conditions, applying ramp-wave protocols (from −150 to +50 mV, 180 ms, holding interpulse potential at −50 mV). KCa3.1 functional expression was quantified by measuring the NS6180-sensitive current at −150 mV and expressed as current density (pA/pF, current divided through whole-cell capacitance). Remaining after NS6180 block whole-cell current was composed of (normally small) non-specific leak and voltage-dependent Kv1.3 currents; leak current was linearly extrapolated to +50 mV and subtracted, yielding Kv1.3 current (expressed in pA/pF).

### 2.5. Activation of Primary NK and NK-92 Cells

Administration of Il-2, Il-15, or their combination with primary NK cell culture ex vivo or NK cell lines was shown to be an effective method of their activation [28,29,30,31]. In this work, for the activation of NK cells, a cocktail of IL-2 (500 UI/mL) and IL-15 (1 ng/mL for primary NK and 0.3 ng/mL for NK-92 cells) was added to the cell culture. Working concentrations were adjusted as minimal ones producing a significant increase in functional expression of the KCa3.1 channel in 24 h. Activated cells were used for electrophysiological and confocal microscopy assays.

### 2.6. Morphological Analysis of Activated Primary NK Cells

Morphological changes were estimated by analysis of cell circularity and cell surface area. Resting primary NK cells were placed in a custom-made chamber (1 × 10^6^/mL) in complete medium and evaluated by confocal microscopy (LSM 700, Carl Zeiss, Jena, Germany). Prior to activation, a period (20 min) was established to allow cell sedimentation. Control images (micrographs of unstimulated cells) were acquired, and a cocktail of IL-2/IL-15 (500 UI/mL and 1 ng/mL) was then applied gently to avoid cell agitation. Images were generated with Zen lite 3.0 software (Carl Zeiss, Jena, Germany). Raw images were further processed using ImageJ 1.53t software (NIH). Briefly, 50 regions of interest (ROI) delimiting individual cells were selected by each micrograph using the ROI manager. Later, the Area/Shape descriptors tools were employed to analyze the characteristics of each selected cell. A minimum of 10 different micrographs from at least 3 independent experiments were evaluated. Raw data was further processed and analyzed employing GraphPad Prism v.8.0 (GraphPad Software, San Diego, CA, USA).

### 2.7. Intracellular Ca^2+^ Measurements

NK cells were loaded with membrane-permeable Fura-2-AM or Fluo-4-AM, both as non-fluorescent acetoxymethyl esters (F1201 and F14201, respectively; Invitrogen by Thermofisher Scientific). Cells were counted and collected (1 × 10^6^/mL; 400× *g*/5 min), centrifuged, and resuspended in Hanks Balanced Salt Solution (HBSS; 143 mM NaCl, 6 mM KCl, 5 mM MgSO_4_, 1.5 mM CaCl_2_, 20 mM HEPES pH 7.4, 0.1%BSA, 5 mM glucose, ≈300 mOsm) with either Fluo-4 or Fura-2 (2 μM for any of the fluorophores). Cells were further incubated for 30 min at room temperature, protected from light. After the incubation, cells were washed and resuspended in either HBSS or Ca^2+^-free HBSS, depending on the experiment type. Prior to the experiment, cells were incubated for 15 min to allow the cleavage of AM-forms by intracellular esterases, which convert Fura-2-AM and Fluo-4-AM to fluorescent Fura-2 and Fluo-4, sensing intracellular Ca^2+^. Representative micrographs of Fluo-4 stained NK cells were acquired by confocal microscopy (LSM700). Fluo-4 fluorescence intensity was estimated either by confocal microscopy by selecting individual cells (ROI manager of ImageJ software) or by flow cytometry (FACS Canto II, FITC channel). Ca^2+^ transients were recorded either by spectrofluorometry (F7000; Hitachi High Technologies, Tokyo, Japan), placing the cells (1 × 10^6^/mL) in a quartz cuvette and maintaining them under constant agitation, or by a fluorescent plate reader (GloMax Discover, PROMEGA, Madison, WI, USA). Raw data was normalized to initial dye fluorescence. To study the effect of NS6180 on Ca^2+^ response, cells were preincubated with the blocker for 20 min. Raw data from at least 4 independent experiments were collected and further analyzed using GraphPad Prism.

### 2.8. Degranulation Assay

The lysosomal-associated membrane protein-1 (LAMP-1, also known as CD107a) is a functional marker of NK degranulation [32] and was considered an indicator of NK cytotoxicity [33]. It is present in the membrane of the intracellular cytotoxic granules in NK cells and is externalized after degranulation. The degranulation after NK activation, either by IL-15 addition or target cell recognition, was assayed by means of confocal microscopy (LSM700) or flow cytometry (FACS Canto II). Briefly, basal CD107a expression was estimated in unstimulated NK or Jurkat cells by adding 5 μL of anti-CD107a antibody conjugated with FITC (Biolegend). NK cells, traced with CellTracker Deep Red^TM^ (50 nM; Ex^MAX^ 630 nm/Em^MAX^ 650 nm; C34565; Invitrogen by Thermo Fisher Scientific), were cocultured for different times (0–4 h) with Jurkat cells (E/T = 1:1). Then, cocultures were evaluated by flow cytometry and the desired population, either NK or Jurkat, was selected. From the gate of desired cells, the mean fluorescence intensity (MFI) of FITC fluorescence (CD107a) was calculated. Data from at least 4 independent experiments were collected and averaged. Degranulation assays were performed in HBSS with monensin (2 μM), a carboxylic ionophore that neutralizes the acidic pH of lysosomes, to prevent the autophagic degradation of the endocytosed/trogocytosed anti-CD107a by constitutive protein recycling by target cells. This strategy was originally developed to enhance the signal/noise ratio of fluorescent approaches to detect cytokines, and it was eventually adapted to the detection of other molecules, either by flow cytometry or microscopy [34]. Representative fluorescent micrographs of cocultures were acquired by confocal microscopy (LSM700) and analyzed employing the ImageJ software.

### 2.9. Cytotoxic Activity of NK Cells

Cytolytic granules of NK cells possess large amounts of perforin, a glycoprotein responsible for pore formation in the plasma membrane of the target cells. Perforin oligomerization allows free and non-selective passive transport of ions, water, and small molecules [35]. Target cells (Jurkat, 1 × 10^6^/mL) were stained with CellTracker Deep Red^TM^ (50 nM; Ex^MAX^ 630 nm/Em^MAX^ 650 nm), and cells were washed and resuspended in HBSS. Perforated target cells release CellTracker Deep Red to the supernatant. Unstained NK cells were added at a 1:1 effector/target ratio, and cells were cocultured for 0, 1, or 4 h. After this, the coculture was centrifuged (400× *g*/5 min), and the supernatant was collected to estimate the amount of CellTracker Deep Red released. This was measured employing the fluorescence plate reader by exciting the samples at 627 nm and collecting the emission at 660–720 nm (GloMax Discover, PROMEGA). Raw data from 12 samples of 3 independent experiments were collected and averaged.

### 2.10. NK Mediated Cell Death

To estimate the NK-mediated target cell killing, the NK cells were stained with Hoechst (10 μM, 20 min; Ex^MAX^ 361 nm/Em^MAX^ 486 nm; Thermo Fisher Scientific), while Jurkat cells were placed in HBSS (2.5 mM Ca^2+^) in the presence of the Dead Cells Apoptosis Kit (Invitrogen from Thermo Fisher Scientific; V13245) including an apoptosis indicator (Annexin V-A488, 2 μL; Ex^MAX^ 499 nm/Em^MAX^ 521 nm) and necrotic marker (Propidium iodide, 0.3 μL; Ex^MAX^ 535 nm/Em^MAX^ 617 nm). Basal cell death was estimated in Jurkat cells monoculture (time 0). After this, NK cells (Hoechst^+^) were added to monitor the NK-mediated cell death of Jurkat cells (Hoechst^-^) during 0–6 h by means of confocal microscopy (LSM700) or flow cytometry (FACS Canto II). Data from at least 10 micrographs/conditions from 4 independent experiments were collected and analyzed for the cell death type.

## 3. Results

### 3.1. Patch-Clamp Analysis of K^+^ Currents in Resting and Activated NK Cells and NK-92 Cell Line

#### 3.1.1. Primary Human Donor NK Cells and the NK-92 Cell Line Functionally Express Kv1.3 and KCa3.1 Channels

**Figure 2:**
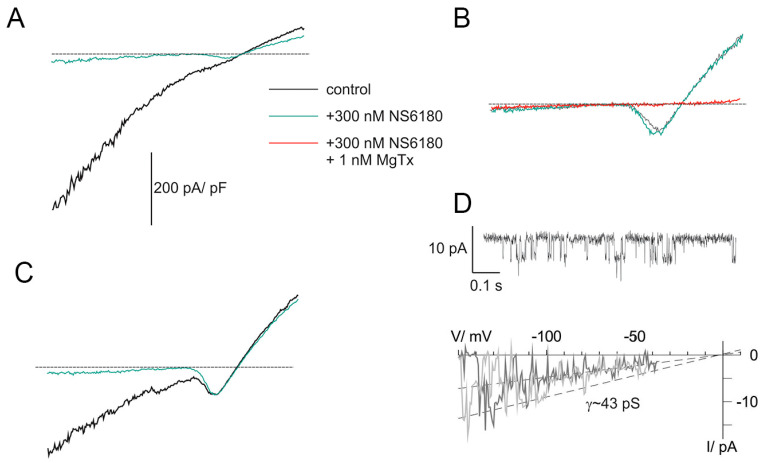
KCa3.1 and Kv1.3 currents in NK cells of healthy donors. (**A**–**C**) Examples of whole-cell current recordings in response to voltage ramps from −150 to +50 mV. A. Activated NK cell, mainly expressing NS6180-sensitive KCa3.1 current (KCa type). (**B**). Resting NK cell expressing only margatoxin-sensitive Kv1.3 current (Kv type). (**C**). Activated NK cells, expressing both KCa.1 and Kv1.3 currents (KCa + Kv). Dashed lines mark zero current level. (**D**). KCa3.1 single channel recording in the whole-cell mode. Steady state record at −100 mV (**top**). Unitary current–voltage relation (**bottom**), leak current was subtracted from recordings, displaying 1 or 2 open KCa3.1 channels. Slope conductance was calculated from linear regression (dashed line).

Previous studies demonstrated that NK cells, similar to B and T lymphocytes, functionally express two types of K^+^-selective channels, Kv1.3 and KCa3.1 [26,36]. Patch-clamp technique in whole-cell configuration was applied to evaluate currents associated with ion channel activity in NK cells. Clamped voltage was changed linearly from −150 to +50 mV. KCa3.1 channels are fully activated by 1 μM free [Ca^2+^]_i_, and their activity is independent of membrane voltage [36] (they are open even at very large negative potentials, e.g., −150 mV). Contrary to that, Kv1.3 currents are activated, passing the threshold of about −40 mV. The recordings were made at almost symmetric conditions when the reversal potential of the K^+^ current was close to 0 mV. Thus, the activation of Kv channels was visualized as a downward bump, which reached its minimum at about −15 mV (where all Kv are open and conduct inward current) followed by a rise and change of the polarity (open channels now conduct outward currents) (Figure 2A–C). At +50 mV, all available Kv and KCa channels are maximally open, and the currents, mediated by these populations, as well as small background (leak) current, are summed up. To reveal the nature of major ion currents present in primary human NK and NK-92 cells, we used a high-affinity blocker of Kv1.3 and a specific blocker of KCa3.1, margatoxin (MgTx, 1 nM) and NS6180 (300 nM), respectively. According to the published data [37,38], these working concentrations will block about 95% of respective currents, which was indeed observed (Figure 2A–C). Examples in Figure 2A–C represent whole-cell current patterns with a different ratio between functional KCa3.1 and Kv1.3 expression. In Figure 2A, KCa3.1 current dominates over Kv1.3, and whole-cell current displays a notable inward rectification, i.e., slope conductance for inward current was higher than for outward one, consistent with previously reported properties of lymphocyte KCa3.1 current under *quasi*-symmetric high-K^+^ concentrations at both membrane sides [36]. Resting NK cells, isolated from healthy donors, sealed almost ideally with the patch electrode: after the KCa3.1 block, only tiny background leak currents remained at voltages below the Kv1.3 activation threshold (e.g., −150 mV). Getting advantage of this and of the fact that most of the resting NK cells displayed only a few NS6180-sensitive channels active at all potentials, we were able to record single channel current–voltage relation in whole-cell mode, using ramp-wave voltage protocol (up to voltages about −40 mV, where Kv1.3 channels started to open). A linear approximation yields single channel conductance of 43 pS (single channel current of about −6.5 pA at −150 mV) at these ionic conditions (Figure 2D), close to 35 pS reported previously [36]. At micromolar [Ca^2+^]_i_ maximal open probability for a KCa3.1 channel is between 0.3 and 0.4 [36], consistent with our steady state recording at −100 mV (Figure 2D). Obtention of high-quality giga-Ohm seals became challenging for activated NK cells and was always challenging for NK-92 cells, either resting or activated (seal obtention efficiency <5%), which may explain the lack of patch-clamp studies, using this cell line as a model. An example of a successful recording of whole-cell current from an NK-92 cell can be found in Appendix C.

#### 3.1.2. Activation of NK Cells Changes Cell Morphology and Strongly Increases the Percentage of Cells Expressing Large KCa3.1 Current

**Figure 3:**
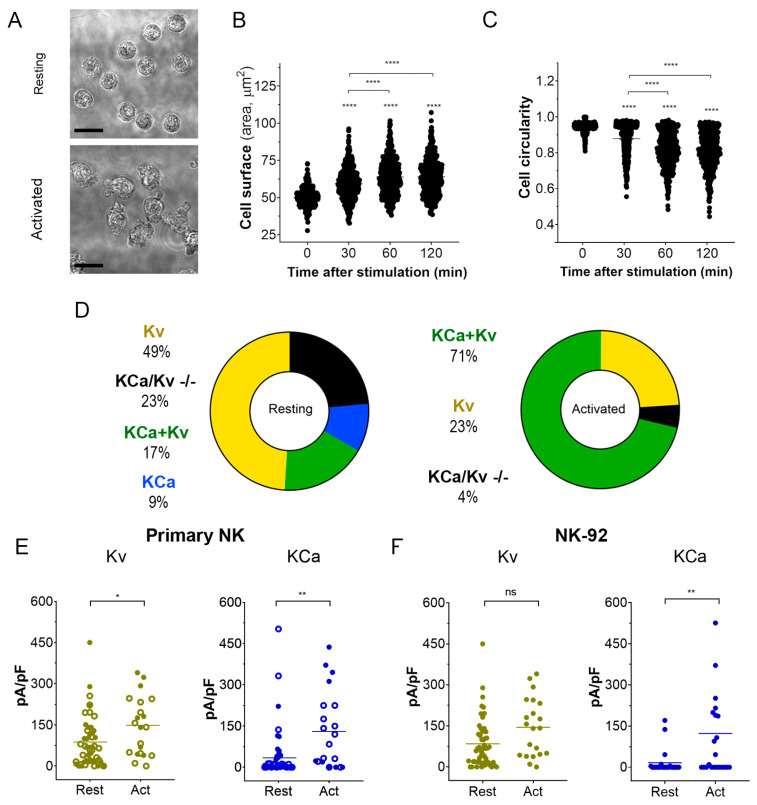
Changes in cell morphology and K^+^ current density upon activation of NK cells. (**A**). Representative micrographs of unstimulated primary NK cells (resting; upper micrograph) and activated primary NK cells (IL-2 and IL-15, 500UI/15 ng/mL respectively; 2 h; lower micrograph). Scale bar, 10 µm. (**B**,**C**). Time course of the change in cell size (**B**) and circularity (**C**) upon primary NK cell activation (*n* = 500 individual cells for each time point). One-way ANOVA was performed, followed by a Tukey’s Multiple comparison test to determine statistical significance between groups (****: <0.0001). (**D**). Relative distribution of KCa, Kv, KCa + Kv, and KCa/Kv −/− whole-cell current patterns in resting and activated NK cells. A significant Kv1.3 or KCa3.1 current presence was considered when the respective current density exceeded 20 pA/pF at −150 mV for KCa3.1 and +50 mV for Kv1.3. NK cells from 4 donors were analyzed. (**E**,**F**). Kv1.3 (olive) or KCa3.1 (blue) current density in resting and activated primary NK cells (**E**) and NK-92 cell lines (**F**). Open and filled circles are for NK cells from 2 different healthy donors. Independent t-test was employed to determine statistical significance (*n* = 51 for resting and *n* = 21 for activated primary NK cells; *n* = 23 for NK-92 either resting or activated. ns: *p* > 0.05, *: *p* < 0.05, **: *p* < 0.01, ****: *p* < 0.0001).

Activation of NK cells from healthy donors by IL-15 produced significant changes in cell morphology and caused an increase in cell membrane surface within 2 h (Figure 3A–C). An independent estimate of cell surface increase comes from membrane capacitance measurements. Capacitance is proportional to the membrane surface (~100 μm^2^/pF), and mean capacitance increased upon the NK activation from 1.88 ± 0.13 pF (*n* = 51) to 3.44 ± 0.46 pF (*n* = 51). Contrary to healthy donors, the size of the NK-92 blasts slightly decreased upon the activation by IL-15, with a capacitance change from 6.51 ± 0.32 pF to 5.61 ± 0.64 pF (*n* = 23). A small (~15%) percentage of resting NK and NK-92 cells expressed large KCa3.1 current (as one in Figure 2A), and most of the analyzed cells either expressed mainly Kv1.3 current or Kv1.3 + KCa3.1 currents of moderate magnitude (Figure 3D–F). The percentage of NK cells from healthy donors and NK-92 cells expressing high KCa3.1 current density greatly (about 3-fold) increased upon the activation by the IL-15 + IL-2 cocktail. Only a moderate increase of Kv1.3 current density upon activation was observed in NK cells from healthy donors, whereas for NK-92 cells, a similar trend was statistically insignificant (Figure 3E,F). Contrary to Kv1.3 current, the distribution of KCa3.1 current density was non-uniform, with a substantial fraction of cells presenting null KCa3.1 current at any condition. Division of a mean whole-cell KCa3.1 current through a single channel current (Figure 2D) yields an average of 55 KCa3.1 channel copies per activated NK cell, which came close to previously reported values [26]. Excluding the samples with no or marginal KCa3.1 activity, the KCa3.1 functional expression varied greatly (Figure 3E), from 15 to 230 active channel copies per cell.

### 3.2. Effect of KCa3.1 Block on Intracellular Ca^2+^ Dynamics in NK Cells and NK-92 Cell Line 

#### 3.2.1. KCa 3.1 Controls Basal [Ca^2+^]_i_ Level in Resting NK Cells

**Figure 4:**
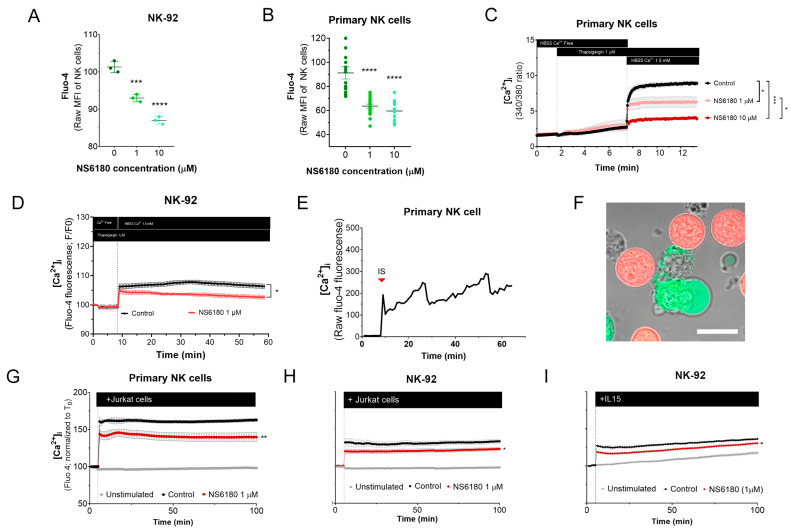
Effect of NS6180 on the basal [Ca^2+^]_i_ level and NK activation. (**A**,**B**). Effect of NS6180 on the basal [Ca^2+^]_i_ levels in NK-92 cells (A, flow cytometry; *n* = 3; 10,000 events per sample) and primary NK cells (B; fluorescent plate reader; *n* > 12; 1 × 10^6^ cells/mL/sample). (**C**,**D**). Effect of NS6180 on SOCE in primary NK (*n* = 4; 1 × 10^6^ cells/mL/sample by spectrofluorometry) and NK-92 cell line (*n* = 3; 1 × 10^6^ cells/mL/sample by fluorescent plate reader). In C, primary NK cells were preloaded with ratiometric Ca^2+^ indicator Fura-2 and fluorescence Ex.340/380 ratio was monitored (Em. 510 nm). In D, NK-92 was preloaded with Ca^2+^ indicator Fluo-4 and fluorescence intensity was measured at Ex: 475 nm, Em: 500–550 nm. (**E**). [Ca^2+^]_i_ oscillations of a primary NK cell after target cell recognition. (**F**) Recognition of target cells (Jurkat, stained with Deep Red) by an NK cell (stained with Fluo-4). (**G**–**I**). NS6180 effects on [Ca^2+^]_i_ of primary NK cells (**G**) or NK-92 cells (**H**) in response to the addition of Jurkat cells or IL-15 (**I**) (*n* = 4). Statistical analysis: (**A**–**C**). One-way ANOVA was performed, followed by a Tukey’s Multiple comparison test to determine statistical significance between groups (***: *p* < 0.001, ****: *p* < 0.0001). (**G**–**I**). Independent *t*-test was employed to determine statistical significance (*: *p* < 0.05, **: *p* < 0.01).

To test the effect of KCa 3.1 blockade in [Ca^2+^]_i_ in NK cells, samples were stained with the Ca^2+^ sensor Fluo 4. Confocal microscopy analysis of individual cells demonstrates that NK-92 cells display diverse Fluo 4 staining patterns, with a relative frequency of spotted > dim > bright (Appendix D). For comparison, we stained with Fluo three T-ALL and one B-ALL cell lines, and in all four cases, the staining was uniform. Preincubation with KCa3.1 specific blocker NS6180 induced a significant reduction in basal [Ca^2+^]_i_ levels in NK-92 cells and primary NK cells (Figure 4A,B). Previous results evidenced that only a small percentage of resting primary NK and NK-92 cells functionally express KCa 3.1 channels (Figure 3D–F). For primary NK cells, there was no significant difference between 1 and 10 μM NS6180 effects, so a respective change in [Ca^2+^]_i_ reflects only the contribution of the cell population fraction, which expresses KCa3.1. At 10 μM NS6180 already exerts a significant (~75%) block of Kv1.3 current [37]. Although working concentrations of drugs with living cells, which actively expulse them, can exceed manyfold working concentrations in vitro, in the case of NK-92 cells, we may not exclude a contribution of Kv1.3 in control of [Ca^2+^]_i_ (Figure 4A).

#### 3.2.2. KCa 3.1 Assists SOCE in NK Cells

NK activation, proliferation, and degranulation are under the control of SOCE, mediated by CRAC. Considering the role of KCa 3.1 in [Ca^2+^] _i_ regulation, we tested the effect of the KCa 3.1 block on SOCE. Applying a standard protocol, which includes Ca^2+^ depletion in ER by thapsigargin in Ca^2+^ free medium, followed by Ca^2+^ supplementation to a final extracellular Ca^2+^ concentration of 1.5 mM, a large increase of [Ca^2+^] _i_ was observed, corresponding to SOCE (Figure 4C). This [Ca^2+^] _i_ response was significantly reduced in the presence of NS6180 (1 μM), while 10 μM almost completely suppressed it. At 10 μM, NS6180 has a significant effect on CRAC and Kv1.3 currents, reducing them by 15% and 75%, respectively [37]. Thus, SOCE in resting primary NK cells greatly depends on KCa 3.1 activity, although a complete abolishment of SOCE may require the block of both KCa3.1 and Kv1.3 channels and a direct effect of NS6180 on CRAC. A similar suppression of SOCE by 1 μM NS6180 was observed in NK-92 cells. The difference with control increased in time, which may reflect the up-regulation of KCa3.1 (Figure 4D).

#### 3.2.3. KCa 3.1 Regulates [Ca^2+^]_i_ Response of NK Cells to Target Cells

A common feature of different activator receptors in NK cells is the downstream phosphorylation of PLCγ, which subsequently mediates the production of IP_3_, activation of IP_3_ receptor channel in the endoplasmic reticulum, resulting in Ca^2+^ depletion of this store, which is coupled to CRAC assembling in the plasma membrane and induction of SOCE. Recognition of Jurkat cell by NK evoked a rapid [Ca^2+^] _i_ peak, followed by long-lasting oscillations (Figure 4E). Upon the formation of the immunological synapse (IS), Fluo-4 fluorescent Ca^2+^ signal was redistributed to the proximity of the IS (Figure 4F). This may be related to the rapid mobilization of KCa3.1 channels to the IS and its colocalization with CRAC, which, in the case of T-cells, resulted in an enhanced Ca^2+^ response [19]. Thus, recognition of a target cell results in a Ca^2+^ rise in the effector cell.

[Ca^2+^] _i_ response of the population of resting NK to Jurkat cells and to IL-15 was significantly reduced by 1 μM NS6180 (Figure 4G–I), indicating that specific block of KCa3.1 in a fraction of NK cells functionally expressing this current has a considerable effect on the global [Ca^2+^]_i_ response.

### 3.3. KCa 3.1 Blockade Optimizes NK Degranulation after Target Cell Recognition

**Figure 5:**
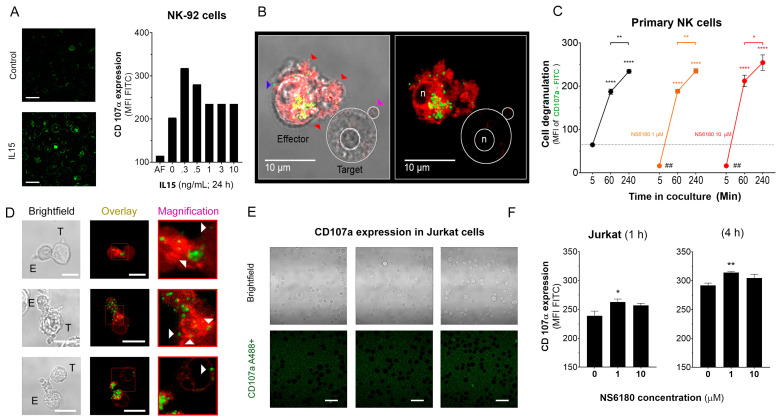
Effect of NS6180 on the degranulation of NK cells. (**A**). Effect of IL-15 (0–10 ng/mL) on NK-92 cell degranulation. Left: Fluorescent images by confocal microscopy show the CD107a surface expression in resting NK-92 cells and after stimulation with IL-15 (0.3 ng/mL, 24 h). Scale bar, 20 µm. Right: representative data of the effect of IL-15 concentration on NK-92 cells degranulation by flow cytometry (10,000 events/sample). (**B**). Example of NK-92 cell degranulation upon Jurkat cell recognition (1 h in coculture, E/T = 1:1). NK-92 cells were stained with Deep Red. Red arrows indicate morphological changes (cells increase their surface, and their shape becomes irregular towards the target cell) induced by the degranulation process. The blue arrow points to a single cytotoxic granule. The pink arrow points to the apoptotic body induced in the Jurkat cell. (**C**). Time course of primary NK cell degranulation upon target cell recognition at control conditions and in the presence of 1 and 10 µM of NS6180. Two-way ANOVA was performed to determine statistical significance (* is used to represent differences within a condition while # is employed to represent differences between conditions; *: *p* < 0.05, **: *p* < 0.01, ****: <0.0001). (**D**). Representative images of CD107a acquisition by target Jurkat cells in contact with primary NK cells. E stays for effector (NK) and T for target (Jurkat) cells. White arrows indicate NK granule delivery to target cells upon IS formation (2 h coculture, scale bar 10 µm). (**E**). CD107a is not expressed at the cell surface of Jurkat cells monoculture (see also Appendix E at larger magnification). Scale bar, 10 µm. (**F**). Flow cytometry analysis of the effect of NS6180 on the CD107a acquisition by Jurkat cells upon 1 h and 4 h in 1:1 coculture with primary NK cells. (*n*:4, One-way ANOVA was performed followed by a Tukey’s Multiple comparison test to determine statistical significance between groups; *: *p* < 0.05, **: *p* < 0.01).

CD107a, also known as LAMP-1, is a marker of intracellular lytic granules in NK cells. NK degranulation can be experimentally traced by measuring the fluorescence intensity of antiCD107 antibody-FITC conjugate, which binds to CD-107a, externalized to the surface of the NK plasma membrane. To validate the assay, NK-92 cells were exposed to different concentrations of IL-15, a well-known NK activator that promotes NK degranulation [39]. Maximal degranulation for NK-92 was observed at an IL-15 concentration of 0.3 ng/mL (Figure 5A). Degranulation was also induced by the presentation of target cells and is reflected by the appearance of bright green puncta on the surface of effector cells (Figure 5B).

Optimal [Ca^2+^]_i_ levels for NK killing activity was close to a resting value of 100 nM, between 122 and 300 nM [6]. Results, presented in Figure 4, demonstrate that block of KCa3.1 limits SOCE and reduces [Ca^2+^] _i_ rise induced by Jurkat cells or IL-15. As expected from the [Ca^2+^] _i_ dependence of the degranulation process, the block of KCa3.1, which resulted in a reduction of [Ca^2+^] _i_, caused an abrupt decrease of degranulation at zero time. However, the degranulation reached the same level as in the control (absence of NS6180) already at 1 h (Figure 5C). Due to a limited reserve of granules, albeit shooting them slower at reduced [Ca^2+^] _i,_ it is expected that sooner or later, the total degranulation reaches the same limit, and it was indeed observed. Following the same logic, a slower shooting seems to be more efficient, potentially hitting more targets consecutively.

The analysis of NK-target cell conjugates demonstrates that upon immunological synapse formation, lytic granules are delivered to Jurkat cells, as evidenced by the presence of multiple fluorescent puncta at the surface of target cells (Figure 5D). Jurkat cells do not express surface CD107a, and there was no CD107a-associated fluorescence in a monoculture of Jurkat cells (Figure 5E). Flow cytometric analysis, gating the target cells of a coculture, evidenced the acquisition of antiCD107a conjugate with A488 by Jurkat cells. Moreover, it was significantly increased in the presence of a KCa 3.1 blocker (Figure 5F). No surface expression of the antiCD107a was observed in Jurkat monoculture upon NS6180 (10 μM) treatment over 2 h (Appendix E). Therefore, most likely, the acquisition of anti-CD107a by target cells reflected a successful granule delivery from NK cells. The surface location of fluorescent puncta in target cells favors the view that Jurkat acquired intact lytic granules by fusion, but we cannot rule out the acquisition of CD107a by trogocytosis. We found it less likely that it was due to higher granules release to a single target cell but due to discharging granules to a larger number of targets. To verify this hypothesis, we tested the effects of NS6180 on Jurkat cell killing.

### 3.4. KCa 3.1 Blockade Improves NK-Mediated Target Cell Killing

**Figure 6:**
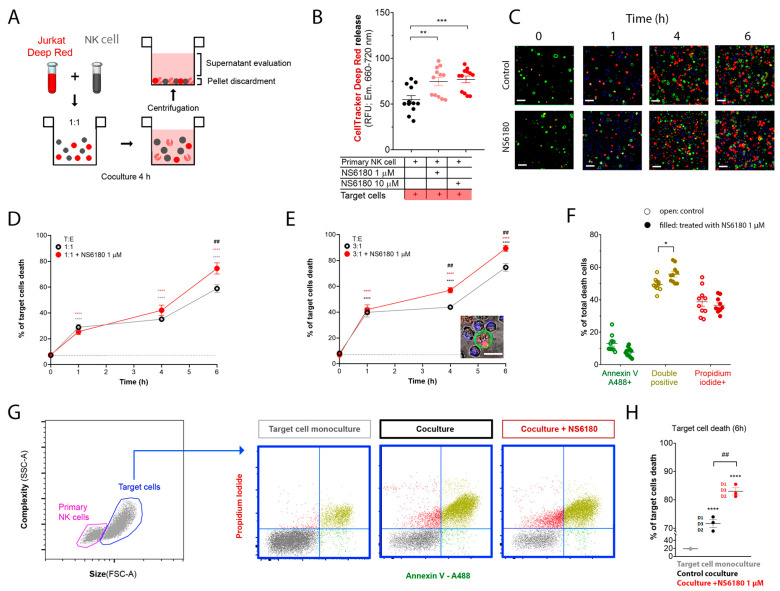
NS6180 improves the NK-mediated killing of Jurkat cells. (**A**). The strategy employed to evaluate NK-induced death of target cells. (**B**). Estimation of Deep Red release by Jurkat cells exposed for 4 h to primary NK cells; data was collected by fluorescent plate reader. (**C**). Representative confocal microscopy images of Jurkat cells cocultured with primary NK cells (1:1) in the absence and presence of 1 μM of NS6180. Blue fluorescence: primary NK cells stained with Hoechst; red: propidium iodide (necrosis); green: annexin-V A488 (apoptosis), scale bar 10 µm. (**D**,**E**). Effect of NS6180 on the time course of Jurkat cells death, induced by primary NK cells in 1:1 and 3:1 T/E ratio. *n* > 2500 cells were analyzed by confocal microscopy for each time point. (**F**). Analysis of Jurkat cell death type induced by primary NK cells at 6 h. Open and filled circles are for the control coculture and NS6180-treated one, respectively (>1000 cells per condition). (**G**). left: Representative dot plot of the characteristics of NK and Jurkat cell populations by flow cytometry. Blue dot plots represent a cell death analysis (*x*-axis: apoptosis, *y*-axis: necrosis) of the target cells gate prior to and after coculture (left and middle, respectively) and in the coculture with NS6180, 1 µM (right). (**H**). Estimation of the NK-mediated Jurkat cells killing by flow cytometry (*n* = 3, 10,000 events/sample). Statistical analysis: (**B**,**D**,**E**,**H**). One-way ANOVA was performed, followed by a Tukey’s Multiple comparison test to determine statistical significance between groups; * was used to indicate a difference from the control, whereas *#* indicates a difference between conditions; *: *p* < 0.05, **: *p* < 0.01, ***: <0.001, ****: <0.0001). (**F**). *t*-test was performed (*: *p* < 0.05).

To monitor the Jurkat cell killing by NK, we loaded target cells with the fluorescent probe CellTracker Deep Red^TM^ (100 nM). The loss of membrane integrity upon cell death results in the release of the dye. Thus, target cells were cocultured with NK for 4 h, and the intensity of CellTracker Deep Red^TM^ fluorescence was measured in the supernatant. This experimental strategy is presented in Figure 6A. KCa3.1 blockade by NS6180 increased the cytotoxic effect of primary NK cells by more than 30% (Figure 6B).

In the next step, cell death analysis was performed. NK cells were stained with Hoechst to distinguish them from target cells, then NK and Jurkat cells at 1:1 and 3:1 ratios were cocultured (0–6 h) in the presence of a necrotic (propidium iodide) and apoptotic (Annexin V-A488) markers. Already at the end of the first hour of coculture, there was a significant increase in necrotic and apoptotic cells number (Figure 6C). The killing was potentiated by 1 μM NS6180 (Figure 6D,E). After 6 h in the coculture with a 3:1 NK/Jurkat ratio, only 10% of Jurkat cells survived vs. 30% in control (Figure 6E). Cell death type analysis by flow cytometry revealed a significant increase of double positive cells, suffering both apoptosis and necrosis (Figure 6F). Global cell death toll in the presence and absence of NS6180 revealed by flow cytometry (Figure 6G,H) was consistent with the confocal microscopy data.

## 4. Discussion

A striking result of the present study is the strong influence of the KCa3.1 blockage on SOCE and target cell-induced Ca^2+^ rise in NK cells (Figure 4). Of note, this result refers to initially resting NK cells. Only a limited fraction of resting NK cells expressed KCa3.1 current, which increased about three times upon NK activation (Figure 3D–F). Thus, during the activation, the effect of the KCa3.1 channel blocker will tend to increase. A large increase of KCa3.1 functional expression upon activation was previously reported for T and B cells [23,36,40,41]’ and SOCE in pre-activated T cells is decreased by about 30% by a selective KCa3.1 blockage or knockout [36,42]. Yet the increase of KCa3.1 current upon activation of B and T cells can be better explained by an almost uniform increase of functional expression in every cell [23,40] contrary to what was observed for NK cells, where a significant fraction of activated ones still displayed low or null KCa3.1 current (Figure 3E,F).

Previously, we have demonstrated that T-ALL leukemic cell lines CCRF-CEM and Molt-4 display a relatively high level of KCa3.1 functional expression, comparable to that for activated T lymphocytes [41]. When stained with Ca^2+^-sensitive fluorophore Fluo-4, CCRF-CEM and Molt-4 cells showed a uniform bright phenotype, contrasting to a rather heterogenous pattern of NK cells staining, where only small (about 20%) fraction displayed a bright appearance (Appendix D). It is tempting to relate this heterogenous pattern to a heterogenous pattern of KCa3.1 expression in resting NK cells, where a comparable small fraction showed a large KCa3.1 current (Figure 3E,F). Another immediate effect of the KCa3.1 blocker was an abrupt decrease in the mean resting Ca^2+^ level in the population of NK cells (Figure 4A,B). Resting [Ca^2+^] _i_ level represents a balance between Ca^2+^ entry, extrusion, and buffering. Therefore, it is conceivable that a reduction of Ca^2+^ entry would move the resting [Ca^2+^] _i_ level to a lower level. However, no significant change in resting [Ca^2+^] _i_ was observed upon KCa3.1 selective blockage in pre-activated T lymphocytes despite that most of them displayed large KCa3.1 current [18]. As only a small fraction (~26%) of resting NK cells expresses KCa3.1 current, but its selective blockage is reduced by ~25% resting Ca^2+^ and by 15–30% Ca^2+^ response of the whole population (Figure 4), the contribution of KCa3.1-expressing cells into the regulation of intracellular Ca^2+^ approaches the theoretical limit. It appears, therefore, that Ca^2+^ homeostasis and homeokinesis are under much stronger influence by KCa3.1 current in NK cells as compared to T lymphocytes.

As expected from the Ca^2+^-dependence of the degranulation, an immediate effect of the reduction of Ca^2+^ entry into NK cells by KCa3.1 blockage was a decrease of degranulation, which has been, however, recuperated in time (Figure 5C). The fact that degranulation at reduced intracellular Ca^2+^ was slowed down will tend to increase the killing efficiency so that NK cells would not discharge their cytolytic granules at the first target but preserve them to hit and kill more target cells [7]. The efficiency of killing was shown to be dependent mainly on temporal rather than spatial organization of degranulation events and, due to the fact that the lethal dose accounts for only 1–2% of total granules content and even though an NK cell releases about 10% of it at a single target, it still can kill multiple cancer cells [43]. Overall, the activity of NK cells, assayed in vitro, could be considered exaggerated. Also, bearing in mind the multiplicity of targets for NK cells, one may presume that the responses to different targets should be different so that the reduction of SOCE in some cases can lead to optimization in some cases but not in others; this requires further detailed investigation.

Our data show that CD107a can be found not only in degranulating NK cells but also in Jurkat cells which most likely reflects the granule delivery to the targets (Figure 5F). Moreover, the presence of a selective KCa3.1 blocker significantly enhanced the uptake of CD107a in target cells. Kinetics of Deep Red accumulation evidenced that the death toll in the presence of NS6180 approaches at 1 h of incubation in control, and after this, the percentage of killed Jurkat cells became significantly higher than in control. Improved killing efficiency was more evident in time for the NS6180-treated NK cells (Figure 6D,E,H). At 1 h, the degranulation in NK cells treated with NS6180 already equals that in control (Figure 5C), but more target cells appeared to be impacted and died in the next hours. Importantly, NS6180 (0–10 μM) did not have any effect on target cell viability at 24 h (Appendix F).

Jurkat cells can be killed by NK cells in two different ways: via degranulation (primarily by necrosis) or through death receptors (by apoptosis), and the two scenarios are often combined. The necrosis/apoptosis ratio increases with the increase of Ca^2+^ entry, indicating that necrosis but not apoptosis depends on external Ca^2+^ [44]. It has been shown for a different target cell model, HeLa, that during serial killing, the degranulation pathway dominates at early times, whereas at later times, NK cells can mediate a single killing event via a slower death receptor pathway [1]. Our data (Figure 6F) evidence the domination of primary necrosis and mixed scenario over apoptosis and that NS6180 tended to increase the necrosis/apoptosis ratio. This can be a consequence of the optimization of the degranulation pathway by control of SOCE.

From a clinical perspective, indirect modulation of SOCE in NK cells by the KCa3.1 block has advantages over direct CRAC suppression, which appears to present enhanced collateral risks. These are related to the widespread tissue distribution of CRAC and the lack of genuinely selective CRAC blockers [11]. KCa3.1 is also expressed in different cell types but, notably, not in excitable tissues [13,45]. A specific block of KCa3.1 by TRAM-34 showed that it is not acutely toxic to multiple cell lines and in vivo in mouse models, even at concentrations 5 μM and above. TRAM-34 did not suppress the activation of resting T-cells but mitogenesis in pre-activated ones [46]. Small synthetic compounds TRAM-34, senicapoc, and NS6180 share the same binding site within the KCa3.1 pore and have very similar mechanisms of action and potency. The most advanced compound, senicapoc, showed promising results in preclinical tests [47]. In turn, NS6180 at sub-micromolar concentrations suppresses the proliferation of splenocytes and secretion of inflammatory cytokines in a rat model [37]. Thus, NS6180 or related drugs appear to be plausible candidates for optimization of cancer cells killing by NK cells, although their immunosuppressive activity needs to be addressed, and it is likely that this treatment should not be applied during other immunotherapy protocols, e.g., CAR-T based ones.

## 5. Conclusions

**Figure 7:**
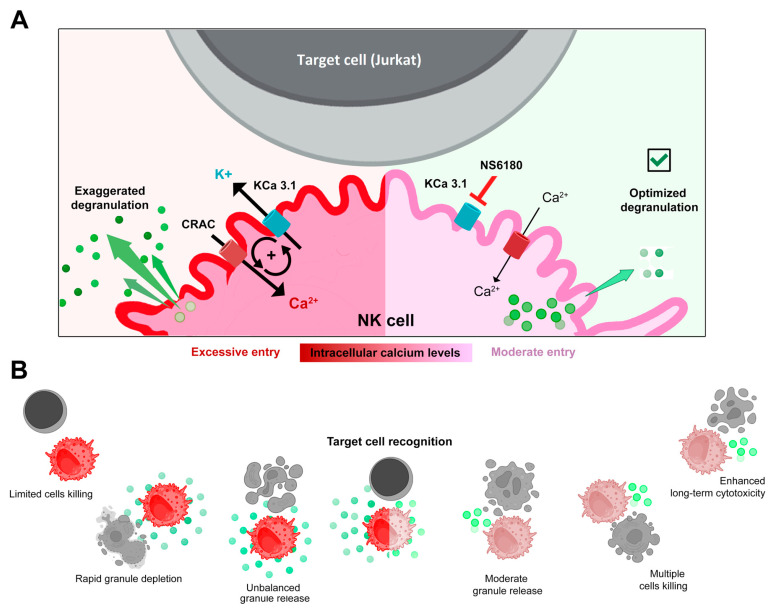
(**A**,**B**) Disruption of the positive feedback between CRAC and KCa3.1 channels by KCa3.1 specific block optimized degranulation and enhanced the killing ability of NK against Jurkat cells. Recognition of a target cell results in the assembly of CRAC/activation of SOCE. This resulted in an increase of [Ca^2+^]_i_ in the effector (NK) cell triggers degranulation, an excessive one in control, and a moderate one in the presence of the KCa3.1 blocker. Ca^2+^-activated KCa3.1 channels mediate K^+^ efflux and hyperpolarization, which in turn potentiates CRAC/ SOCE, thus sustaining high [Ca^2+^]_i_ and high activity of CRAC and KCa3.1 channels. Elimination of this positive feedback loop reduces target-induced [Ca^2+^]_i_ signal and makes the reserve of lytic granules available for longer times, allowing the killing of a greater number of target cells.

Selective blockage of the KCa3.1 channel, which is functionally expressed in ~25% of resting and in ~75% of activated NK cells, strongly reduced SOCE and intracellular Ca^2+^ rise, associated with interleukin (IL-15) activation and target cells (Jurkat) recognition. Block of KCa3.1 current optimized the degranulation time course and improved the killing efficiency of NK against Jurkat cells. A working model based on the findings of the present study is presented in Figure 7.

## Figures and Tables

**Figure A1:**
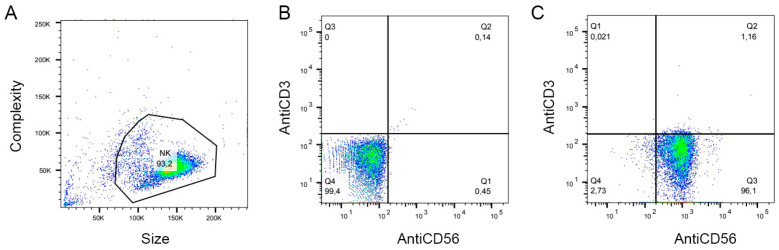
Representative flow cytometry dot plots demonstrating the purity of the primary NK population. (**A**). Gate of the cell population, FSC(Size) vs. SSC (complexity). (**B**). The non-stained sample was used to determine autofluorescence levels (Q4). (**C**). Double-stained (FITC-conjugated anti-hCD56 antibody + PE-conjugated anti-hCD3 antibody) sample: 96% of the cell population are CD3^−^CD56^+^ NK cells (Q1).

**Figure A2:**
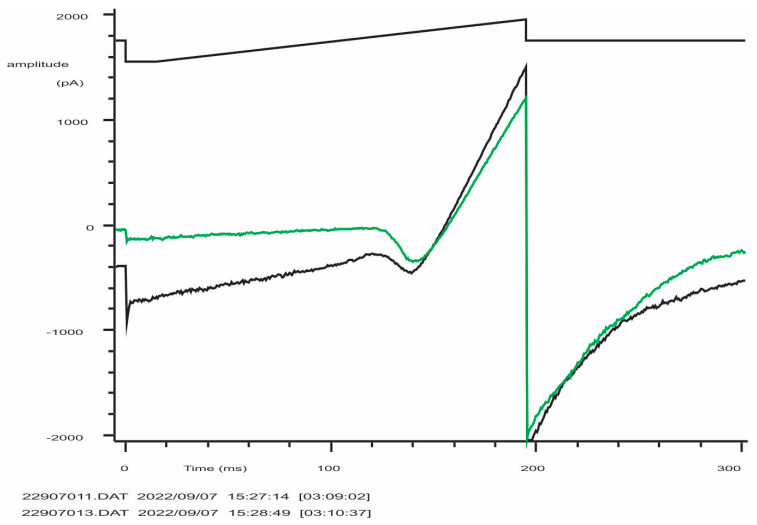
Typical record of K^+^ (Kv and KCa3.1) currents from an activated NK-92 cell (C = 5.5 pF). From a holding potential of −50 mV, the voltage was stepped to −150 mV and then linearly changed to +50 mV, followed by a step to −100 mV (evoking tail current or Kv deactivation). The black trace is the control, green trace is after the application of 300 nM of specific KCa3.1 blocker NS6180. Note that instead of the expected 95% block at −150 mV, an apparent current reduction was by 83% only due to a significant background (leak) current (estimated seal resistance was 1.6 GΩ). The difficulty of obtaining of tight seals with the NK-92 cell line was a constant problem.

**Figure A3:**
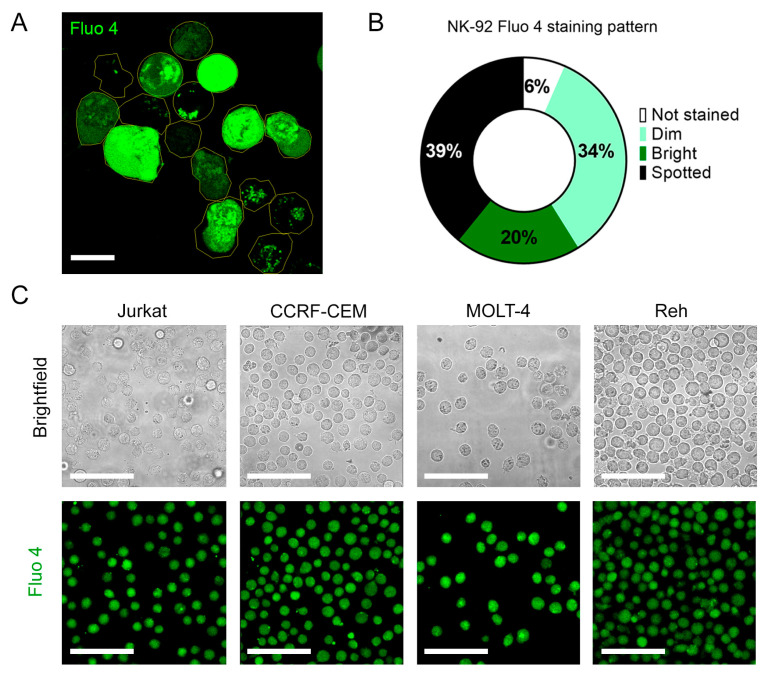
(**A**). Representative confocal micrograph of the heterogenous Fluo-4 staining of unstimulated NK-92 cells. (**B**). Frequency analysis of different Fluo-4 staining patterns for NK-92 cells. (**C**). Fluo 4 staining patterns of T (Jurkat, CCRF-CEM, and MOLT 4) and B (Reh)-ALL cells. Scale bar represents 10 µm for A and 50 µm for C.

**Figure A4:**
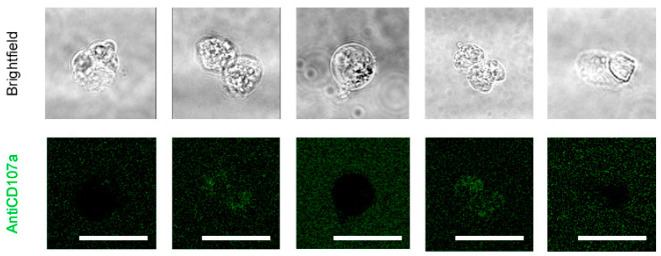
Effect of NS6180 on antiCD107a surface expression in Jurkat cells. Jurkat cells were incubated with NS6180 (10 μM) in the presence of an antiCD107a antibody (green) and monitored for 2 h by confocal microscopy in order to trace the antiCD107a staining. The green fluorescence remained in the background. Scale bar represents for A 10 µm.

**Figure A5:**
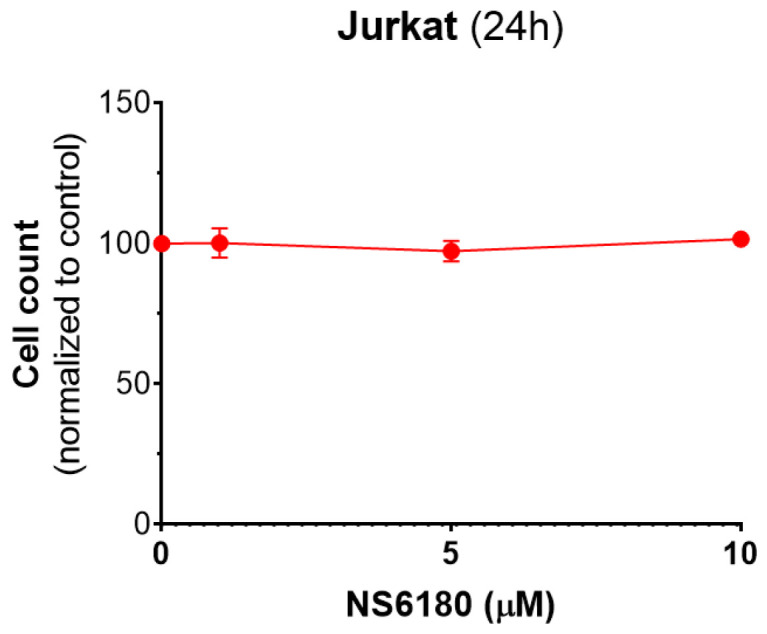
Effect of NS6180 on Jurkat cells viability. In total, 1 × 106 cells/mL were analyzed for each condition, and NS6180 was administered for 24 h prior to the cell count.

## Data Availability

The data presented in this study are available within the article.

## Appendix A

**Table A1:** List of reagents used in this study. NS: not specified. NA: not applicable. Gibco (Billings, MT, USA); Equitech-Bio (Kerrville, TX, USA); Sigma-Aldrich (Saint Louis, MO, USA); Stemcell (CDMX, Mexico, MX); Biolegend (San Diego, CA, USA); Cytiva (Marlborough, MA, USA); Miltenyi Biotec (Auburn, CA, USA); Invitrogen (Waltham MA, USA) and Tocris (Bristol, UK).

Product	Supplier	Catalog/Specification Number	Stock Concentration	Solvent
Culture media components				
RPMI advanced	Gibco	12633-012	1X	NS
HEPES	Gibco	15630-080	1M	H_2_O
GLUTAMAX	Gibco	35050-061	100X	NS
FBS	Gibco	0622	NS	NS
Antibiotic-antimycotic	Gibco	15240-062	100X 10^3^ UI/mL penicillin, 10^3^ μg/mL streptomycin, 25 μg/mL B-amphotericin	NS
αMEM	Gibco	12561-056	1X	NS
Horse serum	Equitech-Bio, Inc.	SE30	NS	NS
Myo-Inositol	Sigma	I7508-59G	100 mM	H_2_O
2-Mercaptoethanol	Sigma	M3148	14.3 M	NS
Folic acid	Sigma	F8758	50 mg/mL	1M NaOH in H2O
IL2	Stemcell	78036.2	1 μg/mL	Sterile water
IL15	BioLegend	570314	20 ng/mL	Sterile water
Reagents for NK isolation				
Ficollpaque plus o histopaque	Cytiva	17144003	NA	NS
NK isolation kit	Miltenyi Biotec	130-092-657	NA	NA
Cell death kit				
Dead Cell Apoptosis Kit	Invitrogen by Thermo Fisher Scientific	V13245	NA	NA
Annexin binding buffer	Invitrogen by Thermo Fisher Scientific	V13246	1x	PBS
Fluorescent dyes and antibodies				
Anti-CD3-PE	Biolegend	300308	NS	NS
Anti-CD56-FITC	Biolegend	362546	NS	NS
Anti-CD107a-FITC	Biolegend	326810	NS	NS
Fluo-4 AM	Invitrogen by Thermo Fisher Scientific	F1221	2 mM	DMSO
Fura-2 AM	Invitrogen by Thermo Fisher Scientific	F14201	2 mM	DMSO
CellTracker Deep Red	Invitrogen by Thermo Fisher Scientific	C34565	2 mM	DMSO
Hoechst 33342	Thermo Fisher Scientific	62249	20 mM	H_2_O
Blockers, salts, buffers				
Monensin	Sigma-Aldrich	22373-78-0	2 mM	Methanol
NS6180	Tocris	4864	10 mM	DMSO
MgTx	Tocris	3563	10 mM	H_2_O
PBS	Gibco	70013-032	10X	H_2_O
CaCl_2_	Sigma	C-7902	1 M	H_2_O
MgCl_2_	Sigma	M-2670-100G	1 M	H_2_O
KCl	Sigma	P-5405	NA	H_2_O
EGTA	Sigma	E-4378	100 mM	H_2_O
HEPES	Sigma	H-7523	NA	H_2_O
KOH	Sigma	P-5958	1 M	H_2_O
NaOH	Sigma	S-0899	1 M	H_2_O
